# Modeling Analysis of Silk Fibroin/Poly(ε-caprolactone) Nanofibrous Membrane under Uniaxial Tension

**DOI:** 10.3390/nano9081149

**Published:** 2019-08-10

**Authors:** Yunlei Yin, Xinfei Zhao, Jie Xiong

**Affiliations:** 1 College of Materials and Textile, Zhejiang Sci-Tech University, Hangzhou 310018, China; 2 School of Textile, Zhongyuan University of Technology, Zhengzhou 450007, China; 3 Key Laboratory of Advanced Textile Materials and Manufacturing Technology of Ministry of Education, Zhejiang Sci-Tech University, Hangzhou 310018, China

**Keywords:** electrospinning, nanofibrous membrane, geometric modeling, uniaxial tensile

## Abstract

Evaluating the mechanical ability of nanofibrous membranes during processing and end uses in tissue engineering is important. We propose a geometric model to predict the uniaxial behavior of randomly oriented nanofibrous membrane based on the structural characteristics and tensile properties of single nanofibers. Five types of silk fibroin (SF)/poly(ε-caprolactone) (PCL) nanofibers were prepared with different mixture ratios via an electrospinning process. Stress–strain responses of single nanofibers and nanofibrous membranes were tested. We confirmed that PCL improves the flexibility and ductility of SF/PCL composite membranes. The applicability of the analytical model was verified by comparison between modeling prediction and experimental data. Experimental stress was a little lower than the modeling results because the membranes were not ideally uniform, the nanofibers were not ideally straight, and some nanofibers in the membranes were not effectively loaded.

## 1. Introduction

Electrospinning is an inexpensive and simple method that is broadly applicable for the controllable production of ultrafine continuous fibers with a high surface-to-volume ratio and high porosity. The tuning and controlling of these properties are often crucial for advanced biomedical applications like wound dressings [1,2], scaffold engineering [3,4], drug delivery devices [5], medical implants [6], and more [7,8].

Among the numerous materials suitable for tissue engineering, silk fibroin (SF) obtained from Bombyx mori silkworms is one of the most widely implemented as a scaffold material for tissue engineering due to its excellent biocompatibility and low immune reaction [9,10]. However, SF fibrous membranes fabricated via electrospinning are brittle due to the formation of a crystalline β-sheet secondary structure. This drawback limits the application of electrospun SF in tissue engineering [11]. Methods of improving SF mechanical performance include blending with other synthetic polymers such as polyethylene oxide (PEO) [12], poly(ε-caprolactone) (PCL) [13], polylactic acid (PLA) [14], polyglycolic acid (PGA) [15], and their copolymers [16,17]. The resulting nanocomposites possess the characteristics of the initial constituents, including the excellent mechanical properties of the polymers and the biocompatibility of SF [18]. SF is often combined with a biodegradable PCL given its outstanding strength and elasticity [19]. One example is preparation of electrospun SF/PCL nanofibrous scaffolds using formic acid (FA) [20] and 1,1,1,3,3,3-hexafluoro-2-propanol (HFIP) [19,21]. Applications of SF/PLC composites include regeneration skin [11], heart [22], bone [23] and vascular tissues [24].

Understanding the mechanics of nanofibrous membranes is important when evaluating their mechanical properties at various structural levels both during processing and use for final applications. Many researchers have tested the mechanical properties of polymer nanofibers and membranes. Ko et al. [25] obtained elastic moduli of carbon nanotube (CNT)/polyacrylonitrile (PAN) nanofibers using the atomic force microscopy (AFM) bending technique. Tan et al. [26] collected tensile strength data for a single electrospun PEO nanofiber using a mobile optical microscope stage coupled with a piezoresistive AFM tip. In another study, the tensile properties of a single-strand PCL electrospun ultrafine fiber were tested using a nano tensile instrument [27]. Lin et al. [28] reported a method based on a stream of air to determine the mechanical properties of electrospun fibers. Other researchers tested tensile properties of nanofibrous membranes [29,30,31,32,33]. The classical methods developed by Petterson [34] and Hearle and Stevenson [35] based on the mechanics of a nonwoven mat are still beneficial for understanding the mechanical properties of nanofibrous membranes. Yin et al. [36] analyzed how the properties of single nanofibers affect corresponding nanofibrous membrane.

Several analytical [37,38], semi-analytical [39,40] and numerical [41,42] methods have been adopted to predict the tensile properties of electrostatic textiles. However, these methods require a relatively complicated calculation. As the main factors affecting the mechanical properties of fibrous membranes are the micro-structure and single fiber properties of fibrous membranes, in this study we established the uniaxial tensile force relationship between single fibers and fibrous membranes using micro-mechanical analysis. Tensile mechanical properties of single fibers were correlated with those of fibrous membranes through a simple mechanical relationship model and the accuracy of the model was analyzed.

In this paper, a geometric modeling analysis is proposed to predict the uniaxial behaviors of randomly oriented nanofibrous membranes. For this purpose, we used the tensile and structural characteristics of single fibers and nanofibrous membrane, respectively. Five types of SF/PCL nanofibers with different mixture ratios were prepared via an electrospinning process. The stress–strain responses of single nanofibers from these nanofibrous membranes were tested for further use in the model. The applicability of the analytical model was examined by a comparison between the modeling prediction result and the experimental data.

## 2. Modeling Analysis

### 2.1. Assumptions

**Figure 1:**
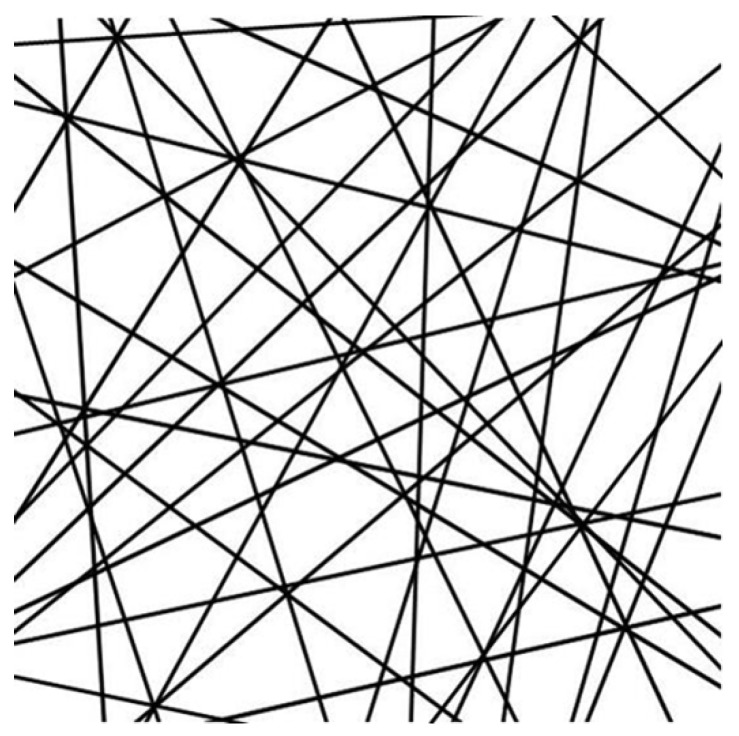
Schematic diagram of electrospun nanofiber membrane.

Firstly, the nanofibers in electrospun membrane were simplified as continuous and straight filaments. The membrane consisted of layers of randomly oriented nanofibers in the in-plane direction, as shown in Figure 1. Secondly, we excluded the interlayer effect. Nanofibers were deposited and randomly overlapped, ideally in sequence, during the whole electrospinning progress. No in-plane adhesion among fibers was considered. Therefore, the mechanical response of every nanofiber was assumed to be independent. Thirdly, time-dependent properties were not considered. The stretching speed was controlled to ensure that the specimens were under quasi-static tensile (0.002 s^−1^). Neither the strain rate effect nor the stress relaxation were considered.

### 2.2. Derivation

**Figure 2:**
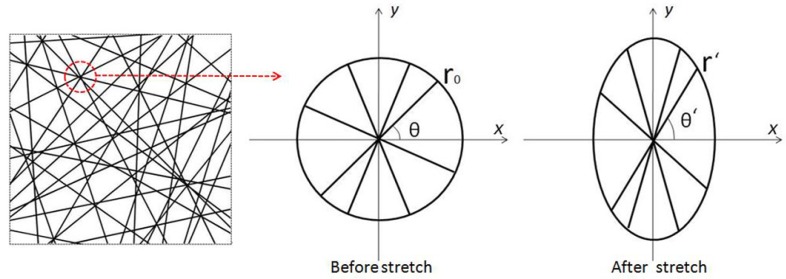
Changing of fiber’s orientation under stretch.

The membrane consists of layers of randomly oriented nanofibers. Many intersections exist where fibers meet. As shown in Figure 2, a circle is used to represent a unit intersection of the membrane, and some randomly-oriented fibers pass through the center of the circle with the same length 2 $r_{0}$. Every fiber has its own angle (*θ*) of inclination. If a stretch along the *y* axis is applied, the circle becomes an ellipse after tensile stretching. Then, the angle of inclination (*θ*) becomes $\theta^{\prime }$ and the length of fiber (2 $r_{0}$) becomes 2 $r^{\prime }$. The stretch of the inclined fiber is coordinated with the deformation of the whole circle.

Therefore, the strain of the membrane is described as follows:

(1) $$\varepsilon =\frac{r^{\prime }sin\theta^{\prime }-r_{0}sin\theta }{r^{\prime }sin\theta^{\prime }}$$

Due to the large deformation during stretch progress, the instant $\theta^{\prime }$ is obtained by

(2) $$\theta^{\prime }=arctan\frac{\left(1+\varepsilon \right)sin\theta }{vcos\theta }$$

where v is the shrinkage coefficients of fiber membranes, $v=\frac{\varepsilon_{x}}{\varepsilon_{y}}$. The deformed fibers are preferentially distributed along the stretching direction and fibrous membranes shrink horizontally. During tensile deformation, the shrinkage coefficient v of fibrous membranes is critical to the analysis of the deformation of nanofibers. Based on Petterson [43], since fibers are rearranged when stretching and the probability density function of the orientation distribution of fibers is difficult to calculate, the deformation calculation of single fibers is complicated. Therefore, it is feasible to determine the deformation of fibers by measuring and calculating the longitudinal and transverse deformations of fibers during stretching.

**Figure 3:**
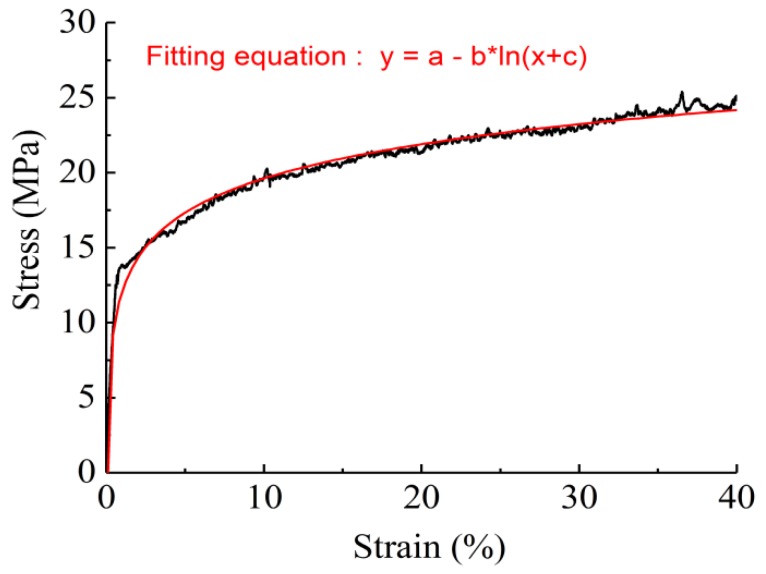
Typical stress–strain response of single nanofiber and its fitting equation.

The force in the single fiber along the y axis by the applied loading is described as follows:

(3) $$f_{y}=\frac{\pi }{4}d^{2}\sigma_{f}sin\theta^{\prime }f_{y}=\frac{\pi }{4}d^{2}\sigma_{f}sin\theta^{\prime }$$

where *d* is the diameter of the fiber and $\sigma_{f}$ is the axial stress along the fiber’s orientation. However, since the diameters of nanofibers are not uniform, the stress–strain data obtained from the single nanofiber tests should not be used directly to predict the mechanical behaviors of membranes. Therefore, the mathematical fitting, as shown in Figure 3 and Table 1, was applied to obtain stable results from the test data.

Then the harmonic stress $\bar{\sigma_{f}}$ is based on the variation in the diameters of the nanofibers in membrane as follows:

(4) $$\bar{\sigma_{f}}=\frac{1}{2}\left(1+\frac{d}{D}\right)\left[a-b\;ln\left(\varepsilon +c\right)\right]$$

where *a*, *b*, and *c* are fitting parameters, and *D* is the harmonic average of the diameters of the nanofibers. The fibers in the membrane are randomly oriented. Therefore, the integral algorithm was used to obtain the average force $\bar{f_{y}}$ along the y axis from every fiber with oriented angle from 0 to π/2 as follows:

(5) $$\bar{f_{y}}=\frac{2}{\pi }\int_{0}^{\frac{\pi }{2}}f_{y}d\left(\theta \right)$$

Finally, the total force $F_{y}$ along the y axis from all fibers is described as

(6) $$F_{y}=n\bar{f_{y}}$$

(7) $$n=\frac{2V\left(1-p\right)}{r_{0}\pi D^{2}}$$

where *n* is the amount of fibers in unit volume *V* and *p* is the porosity of the electrospun membrane.

## 3. Materials and Methods

### 3.1. Materials

SF/PLC spinning dope solution was prepared using regenerated SF sponge and PCL (with molecular weight of ~80,000 g∙mol^−1^, Guanghua Weiye Co., Ltd., Shenzhen, China). Other materials (Na_2_CO_3_, CaCl_2_ and 99% methanol) were obtained from Hangzhou Gaojing Fine Chemicals Co., Ltd. (Hangzhou, China). Formic acid (FA. 98.0% pure) was purchased from Shanghai Lingfeng Chemical Reagent Co., Ltd. (Shanghai, China). All materials were used as received without any additional treatment.

### 3.2. Preparation of Stock SF Sponge

Silk cocoons were boiled in 0.05 vol% Na_2_CO_3_ solution for 30 min, after which they were rinsed with distilled water and dried at 40 °C overnight. After that, these degummed silk threads were placed into a 1:8:2 mixture (by moles of CaCl_2_, H_2_O, and EtOH) at 75 °C for 5 min. After the silk threads dissolved, they were dialyzed using a cellulose 12–14 kDa tubing. Distilled water was used as counter-solution. Dialysis lasted for 3 days. The resulting aqueous SF solution was removed from the dialysis tubing, filtered and freeze dried. The resulting materials were regenerated SF sponges, which were stored in a desiccator prior to their use.

### 3.3. Preparation of SF/PCL Solutions

Mixtures containing regenerated SF sponges and PCL at 100/0, 75/25, 50/50, 25/75, and 0/100 weight ratios were dissolved in 18 wt % FA solution and maintained at room temperature for 2 h. After that, the mixtures were stirred for 3 h.

### 3.4. Preparation of SF/PCL Nanofibrous Membranes

A syringe with a stainless-steel needle (22 G) containing 10 mL of spinning dope solution was placed into a special pump. Electrospinning conditions were 0.6 mL·h^−1^ feed rate and 15 kV voltage applied between the needle tip and an aluminum sheet collector located 10 cm away from the needle and mounted on the vertical metal mesh surface. Relative humidity was maintained below 50% during electrospinning.

### 3.5. Morphology of the Prepared Nanofibrous Membranes

**Figure 4:**
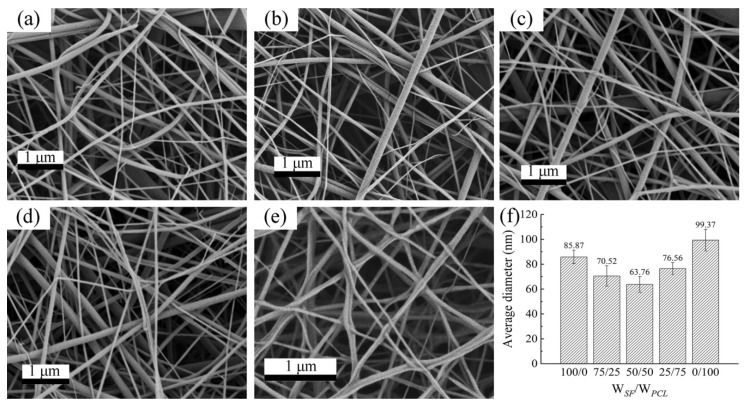
Field emission (FE)-SEM micrographs of the five different types of electrospun membranes: (**a**) W*_SF_*:W*_PCL_* = 100:0, (**b**) W*_SF_*:W*_PCL_* = 75:25, (**c**) W*_SF_*:W*_PCL_* = 50:50, (**d**) W*_SF_*:W*_PCL_* = 25:75, and (**e**) W*_SF_*:W*_PCL_* = 0:100; and (**f**) average diameter of nanofibers.

Nanofiber morphologies were characterized by a Carl Zeiss (Oberkochen, Germany) field emission scanning electron microscopy (FE-SEM) instrument operated at 3 kV accelerated voltage. A thin gold layer was sputtered on the fibers to improve their conductivity. The average diameter was calculated based on 100 measurements of different fibers, which were recorded using Image-Pro Plus 6.2 software (ICube, Crofton, MD, USA) using FE-SEM images. Micrographs and diameters of nanofibers from five different kinds of membranes are presented in Figure 4 and Table 2, respectively.

### 3.6. Tensile Test for Single Nanofiber and Nanofibrous Membranes

**Figure 5:**
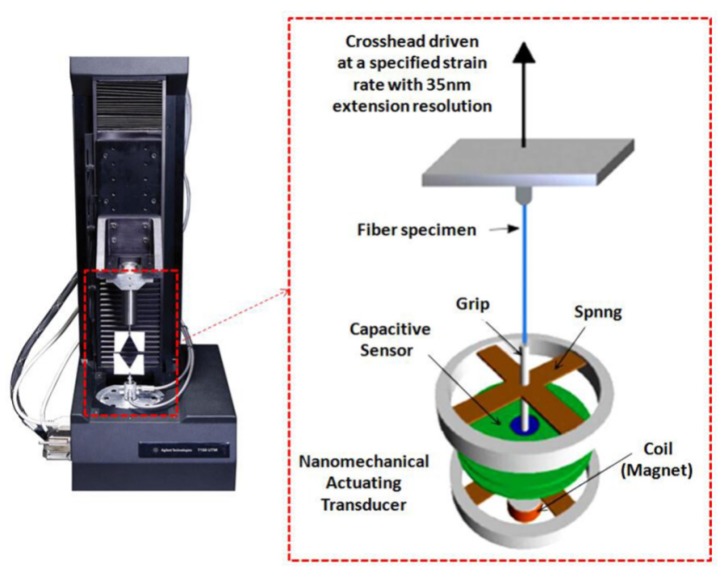
Tensile testing system for single nanofibers.

Stress along the axial of a single nanofiber was tested using a nano-mechanical stretching system (Agilent UTM T150, Santa Clara, CA, USA), which provides excellent mechanical characterization at the nano-scale due to its unique actuating transducer that is capable of generating tensile force load on individual fibers by electromagnetic actuation combined with a precise capacitive gauge (Figure 5).

**Figure 6:**
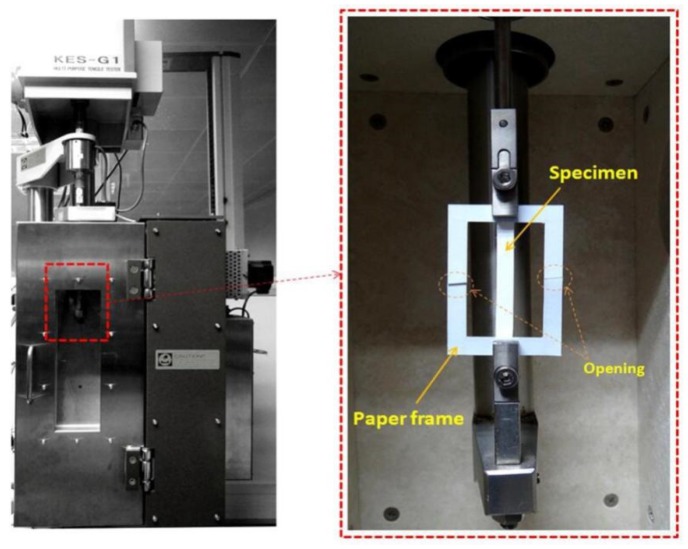
Tensile testing system for nanofibrous membranes.

The uniaxial stress–strain curve of the nanofibrous membranes were tested using the KES-G1 tensile system (Kato-Tech Company, Kyoto, Japan) as shown in Figure 6. Specimens were fixed by two overlap-pasted frames composed of paper to avoid incline and torsion. Before testing, the left and right arms of the paper frames were cut to avoid affecting the stretching of the specimen during tensile testing.

## 4. Results and Discussion

**Figure 7:**
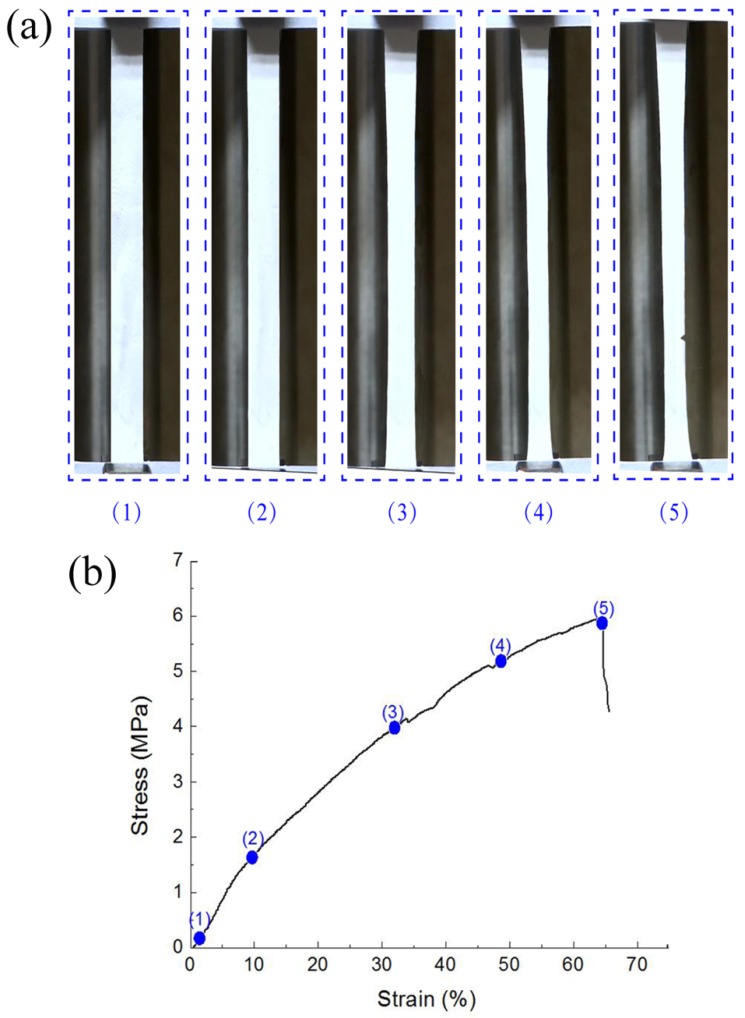
Typical deformation characteristics of nanofibrous membrane with the progress of stress–strain response under uniaxial tensile testing: (**a**) morphology changes of nanofibrous membranes, (**b**) stress–strain curves of nanofibrous membranes.

Figure 7 demonstrates the typical deformative characteristics of nanofibrous membranes under uniaxial tensile testing. Membranes were stretched until fracture occurred. We observed that the rectangle specimen was forced into a dog-bone shape due to tension in the membrane before fracture. The specimen catastrophically failed with a triangular cracking area from the edge of the membrane, which indicated the ductile deformation of nanofibers in the membrane.

**Figure 8:**
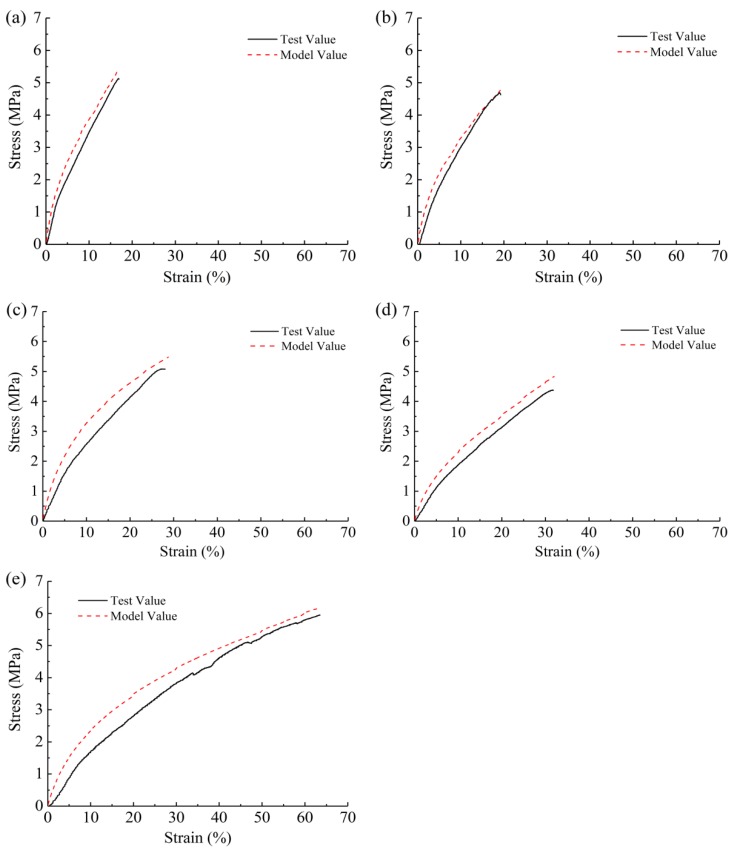
Results of the modeling analysis and experimental tests: (**a**) W*_SF_*:W*_PCL_* = 100:0, (**b**) W*_SF_*:W*_PCL_* = 75:25, (**c**) W*_SF_*:W*_PCL_* = 50:50, (**d**) W*_SF_*:W*_PCL_* = 25:75, and (**e**) W*_SF_*:W*_PCL_* = 0:100 membranes.

Figure 8 shows the uniaxial stress–strain curves of the five different types of SF/PCL membranes. Post-fracture behaviors were not retained for all curves. We found that the strain of membranes increased with increasing PCL content in nanofibers. This indicates that PCL helped to improve the flexibility and ductility of the composite membranes, and that the membranes’ strengths were not obviously affected. These properties are critical for materials used for engineering of vascular and skin tissues.

By comparing the modeling prediction and experimental data, we found that the analytical model is somewhat applicable. The proposed modeling prediction demonstrated similarity to the testing results, showing that the mechanical behaviors of membranes could be predicted based on their structural characteristics and the properties of single nanofibers. However, the experimentally recorded stress values were a little lower than the modeling values. The morphology of the membranes could explain this difference. Firstly, the nanofibrous membranes were not ideally uniform, which could lead to the existence of some weak regions. Secondly, nanofibers were not ideally straight, which could lead to a slow initial increase in stress due to the adjustment of the nanofibers themselves. Thirdly, some nanofibers in membranes were potentially not loaded during tensile process.

## 5. Conclusions

SF/PCL nanofibrous membranes were produced using an electrospinning technique using formic acid. The presence of PCL helped to improve the flexibility and ductility of the membrane without compromising the strength.

In this study, we proposed a geometric modeling analysis based on the tensile properties of single fibers and the structural characteristics of nanofibrous membranes. The applicability of the analytical model was verified by a comparison between the model prediction and experimental data. The experimentally recorded stress level was a little lower than the modeling results for three reasons: (1) the nanofibrous membranes were not ideally uniform, (2) the nanofibers were not ideally straight, and (3) some nanofibers in the membranes were not effectively loaded.

## Figures and Tables

**Table 1:** Parameters obtained from the fitting of single nanofibers.

Type	*a* ^a^	*b* ^a^	*c* ^a^	*R* ^2 b^
SF/PCL = 100/0	27.207	−3.297	1.006 × 10^−4^	0.984
SF/PCL = 75/25	22.669	−2.810	2.663 × 10^−4^	0.976
SF/PCL = 50/50	26.576	−4.313	1.290 × 10^−3^	0.975
SF/PCL = 25/75	12.569	−1.802	5.822 × 10^−4^	0.949
SF/PCL = 0/100	17.130	−3.219	8.120 × 10^−3^	0.975

^a^ The fitting equation parameters for typical stress–strain response of single nanofiber were obtained from Supporting Information Figure 3. ^b^ Coefficient of determination.

**Table 2:** Nanofiber diameters in membranes.

Type	Arithmetic Average Diameter (nm)	Root-Mean-Square Diameter (nm)	Harmonic Average of Diameter (nm)
SF/PCL = 100/0	85.87	91.29	79.49
SF/PCL = 75/25	70.52	76.12	62.96
SF/PCL = 50/50	63.76	71.91	57.11
SF/PCL = 25/75	76.56	80.41	70.54
SF/PCL = 0/100	99.37	110.18	88.62
